# Stress Ulcer Prophylaxis in Mechanically Ventilated Patients With Acute Myocardial Infarction

**DOI:** 10.1016/j.jacadv.2023.100750

**Published:** 2023-12-06

**Authors:** Soumya Banna, Christopher Schenck, Andi Shahu, Alexander Thomas, Cory Heck, Rosanna Tangredi, Tariq Ali, P. Elliott Miller

**Affiliations:** a Department of Internal Medicine, Yale School of Medicine, New Haven, Connecticut, USA; b Section of Cardiovascular Medicine, Yale School of Medicine, New Haven, Connecticut, USA; c Heart and Vascular Medicine, Yale New Haven Hospital, New Haven, Connecticut, USA

**Keywords:** acute myocardial infarction, cardiogenic shock, cost, mechanical circulatory support, outcomes, stress ulcer prophylaxis

## Abstract

**Background:**

Proton pump inhibitors (PPIs) and histamine type 2-receptor blockers (H2Bs) are commonly used for stress ulcer prophylaxis among patients requiring invasive mechanical ventilation (IMV). Recent studies suggest an increased mortality associated with PPIs compared to H2Bs, but these studies poorly represent patients with cardiovascular disease or acute myocardial infarction (AMI).

**Objectives:**

The aim of this study was to compare outcomes related to stress ulcer prophylaxis with PPIs compared to H2Bs in patients with AMI requiring IMV.

**Methods:**

We queried the Vizient Clinical Data Base for adults aged ≥18 years admitted between October 2015 and December 2019 with a primary diagnosis of AMI and requiring IMV. Using multivariable logistic regression, we assessed for the association between stress ulcer prophylaxis and in-hospital mortality.

**Results:**

Including 11,252 patients with AMI requiring IMV, 66.7% (n = 7,504) received PPIs and 33.3% (n = 3,748) received H2Bs. Age, sex, and the proportion of patients presenting with ST-segment elevation myocardial infarction or cardiogenic shock were similar between groups (all, *P* > 0.05). Compared to PPIs, patients receiving H2Bs had a lower mortality (41.5% vs 43.5%, *P* = 0.047), which was not statistically significant after multivariate adjustment (odds ratio 0.97; 95% confidence interval: 0.89-1.06, *P* = 0.49). In unadjusted and adjusted analyses, H2Bs use was associated with fewer ventilator days, less ventilator-associated pneumonia, and lower hospitalization cost but similar *Clostridium difficile* infections.

**Conclusions:**

Among patients with AMI requiring IMV in this observation cohort study, there was no difference in mortality among patients receiving H2Bs vs PPIs for stress ulcer prophylaxis despite fewer ventilator days and lower ventilator-associated pneumonia in those receiving H2Bs.

Stress ulcer prophylaxis is commonly prescribed for critically ill patients to minimize the morbidity related to clinically significant gastrointestinal (GI) bleeding events.^1^ Current guidelines recommend stress ulcer prophylaxis for those at high risk of GI bleeding, which commonly includes patients requiring invasive mechanical ventilation (IMV), coagulopathy, sepsis, shock, history of previous GI bleeding, and those on antiplatelet therapy.^2^, ^3^, ^4^, ^5^ Proton pump inhibitors (PPIs) and histamine-2 receptor blockers (H2Bs) are the most frequently prescribed agents for acid suppression therapy.^6^^,^^7^ In a meta-analysis of 57 randomized controlled trials, including over 7,000 patients, PPIs were found to be more effective at preventing GI bleeding, but potentially increased the risk of pneumonia compared to H2Bs.^8^ More recently, the PEPTIC (Proton Pump Inhibitors vs Histamine-2 Receptor Blockers for Ulcer Prophylaxis Treatment in the Intensive Care Unit) trial of over 26,000 mechanically ventilated patients similarly found those receiving PPIs had a lower risk of upper GI bleeding compared to those randomized to H2Bs. However, 90-day mortality was nonsignificantly (*P* = 0.054) higher in the PPI group and significantly increased in those who underwent cardiac surgery.^9^

Patients presenting with acute myocardial infarction (AMI) may be especially vulnerable to GI bleeding due to the use of antiplatelet and antithrombotic medications,^10^^,^^11^ especially those who are critically ill requiring IMV.^12^ In addition, stress ulcer prophylaxis selection may be particularly critical in this group as PPIs generally have more interactions than H2Bs with antiplatelet and antithrombotic therapy.^13^ A recent scientific statement from the American Heart Association currently recommends PPIs for high-risk patients as “reasonable to administer.”^4^ However, the evidence for either option in this patient population is limited and must be balanced with known risks, including *Clostridium difficile* infections (CDIs) and ventilator-associated pneumonia (VAP).^14^^,^^15^ Given these gaps in the literature, we aimed to compare outcomes related to PPI and H2B use for stress ulcer prophylaxis among mechanically ventilated patients with AMI.

## Methods

### Data source and study population

We queried the Vizient Clinical Data Base (used with permission of Vizient, Inc. All rights reserved), which includes over 1,000 hospitals from 47 U.S. states. Over 97% of academic medical centers and their affiliated hospitals are included in the database. The Vizient Clinical Data Base includes administrative, financial, and pharmacy-related inpatient information.^16^ We included all adults aged ≥18 years admitted between October 2015 and December 2019 with a primary diagnosis of AMI (ST-segment elevation myocardial infarction [STEMI] and non-STEMI) and requiring IMV. Patients who received only a PPI or only an H2B were included in the study cohort. PPIs included pantoprazole, omeprazole, esomeprazole, and lansoprazole and H2Bs included famotidine and ranitidine. Those with GI bleeding on admission (n = 664) and patients who underwent an esophagogastroduodenoscopy (EGD) or colonoscopy (n = 122) before intubation were excluded. To better evaluate outcomes among nonsurgical patients with AMI, patients who underwent coronary artery bypass grafting (n = 2,113), heart transplantation (n = 8), and left ventricular assist device implantation (n = 750) were also excluded. All data obtained from Vizient were deidentified and exempt from the Yale University Institutional Review Board review.

### Variables of interest

Demographic variables included age, gender, race and ethnicity, primary payer, region, bed size, Association of American Medical Colleges-teaching status, and smoking history. Comorbidities included coronary artery disease, previous percutaneous coronary intervention (PCI), previous coronary artery bypass grafting, previous AMI, diabetes, peripheral vascular disease, heart failure, dyslipidemia, hypertension, valvular disease, chronic pulmonary disease, chronic liver disease, end-stage renal disease, obesity, and dementia. The primary admission diagnosis was STEMI and non-STEMI for all patients. Other diagnoses, coded as present on admission, included cardiogenic shock and cardiac arrest.

Identified procedures included left and right heart catheterization, PCI, intra-aortic balloon pump, percutaneous ventricular assist device, extracorporeal membrane oxygenation, heart transplantation, durable left ventricular assist device, noninvasive ventilation, renal replacement therapy, and tracheostomy. Procedures were defined as occurring before or the same day of IMV (defined as before) or after IMV (defined as after). Supplemental Table 1 lists the International Classification of Diseases-10th Revision-Clinical Modification (ICD-10-CM) codes used to identify procedures. Medications and blood product utilization as well as timing of first use were similarly defined as occurring before or the same day of IMV or after IMV.

### Outcomes

The primary outcome of interest was in-hospital mortality. Secondary outcomes included discharge status (home, home with services, skilled nursing/rehab, against medical advice, other, hospice, and expired), total hospital cost, ventilator days, hospital and intensive care unit (ICU) length of stay, CDI, VAP, use of endoscopy (both EGD or colonoscopy after intubation), and blood product use after intubation. To ensure outcome diagnoses occurred during the hospital admission, CDI and VAP episodes were excluded if coded as present on admission. The sensitivity, specificity, and positive predictive value of ICD-10 for CDI have previously been reported at 35.6%, 99.9%, and 79.2%, respectively.^17^ However, there are limited data on VAP ICD-10 coding accuracy.

### Statistical analysis

Baseline characteristics were compared between patients receiving a PPI vs a H2B. Continuous variables were described as mean ± SD and categorical variables were described as frequencies and percentage. The *t*-test was used to compare continuous variables and chi-squared test for categorical variables. Using multivariable logistic regression, we assessed for the association between stress ulcer prophylaxis type (PPI vs H2B) and in-hospital mortality. Covariates used in the multivariable model were purposefully selected as variables known to be associated with acuity as well as risk factors for GI bleeding,^3^ and included age, cardiogenic shock on admission, cardiac arrest on admission, acute renal failure on admission, chronic liver disease, and any mechanical circulatory support device or vasoactive medication use before intubation. Poisson regression (expressed as an incidence rate ratio [IRR]) was used to evaluate length of stay and ventilator days. A gamma regression model was used to evaluate adjusted total hospital costs.

In addition, we performed subgroup and sensitivity analyses. First, since pantoprazole and famotidine are the most common PPI and H2B, we assessed for the association between in-hospital mortality with only patients that received pantoprazole compared to famotidine. Second, we performed an inverse probability treatment weighting (IPTW) analysis to minimize unmeasured confounders. To ensure covariate balance, we assessed weighted standardized differences with a target difference <0.10. All analyses were performed on STATA 16.0 (Stata Corp) with statistical significance considered at a 2-tailed *P* < 0.05.

## Results

### Patient characteristics

We identified 11,252 patients with AMI requiring IMV, 66.7% (n = 7,504) of which received a PPI and 33.3% (n = 3,748) received an H2B (Central Illustration). Baseline characteristics stratified by stress ulcer prophylaxis type are shown in Table 1. Age, sex, and the proportion of patients presenting with either STEMI or cardiogenic shock was not statistically different between groups (all, *P* > 0.05). Compared to those receiving PPIs, patients receiving H2Bs were more likely to present with cardiac arrest on admission (21.0% vs 17.5%, *P* < 0.001). Those who received H2Bs were less likely to have a history of coronary artery disease and end-stage renal disease, but more likely to have a history of hypertension and stroke compared to those who received PPIs (all, *P* < 0.05). The proportion of patients with heart failure, chronic pulmonary or liver disease, cancer, and peripheral vascular disease were not different between groups (all, *P* > 0.05).

**Central Illustration:**
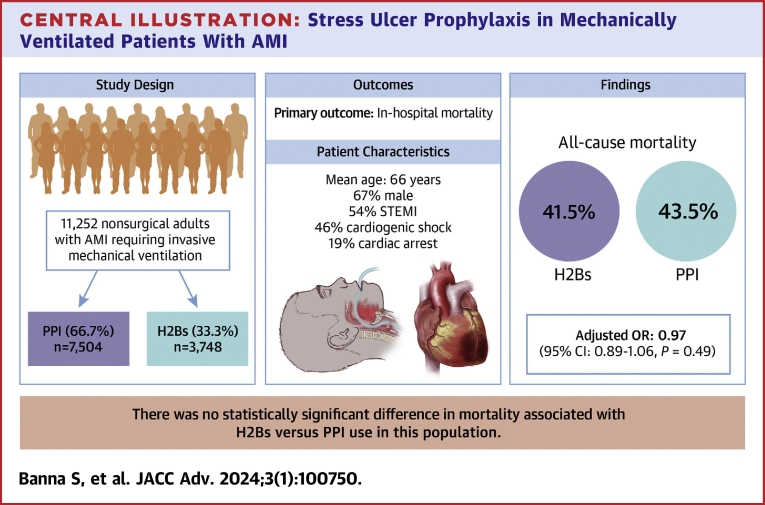
**Stress Ulcer Prophylaxis in Mechanically Ventilated Patients With AMI** AMI = acute myocardial infarction; H2B = histamine type 2 receptor blockers; IMV = invasive mechanical ventilation; PPI = proton pump inhibitors; STEMI = ST-segment elevation myocardial infarction.

**Table 1:** Baseline Characteristics Stratified by Stress Ulcer Prophylaxis

	Stress Ulcer Prophylaxis		
	PPI (n = 7,504)	H2B (n = 3,748)	*P* Value
Demographics			
Age	66.4 ± 12.4	66.0 ± 12.7	0.13
Male	5,015 (66.9%)	2,506 (66.9%)	0.99
Race/ethnicity			
Black	1,244 (16.6%)	702 (18.7%)	0.003
Hispanic	412 (5.5%)	162 (4.3%)	
Other	930 (12.4%)	453 (12.1%)	
White	4,918 (65.5%)	2,431 (64.9%)	
Primary payer			
Commercial	1,654 (22.0%)	926 (24.7%)	<0.001
Medicaid	906 (12.1%)	513 (13.7%)	
Medicare	4,329 (57.7%)	2,036 (54.3%)	
Other	615 (8.2%)	273 (7.3%)	
Admission characteristics			
AAMC teaching hospital	6,234 (83.1%)	2,823 (75.3%)	<0.001
Region			
Midwest	2,248 (30.0%)	1,212 (32.3%)	<0.001
Northeast	1,677 (22.3%)	932 (24.9%)	
South	2,701 (36.0%)	1,245 (33.2%)	
West	878 (11.7%)	359 (9.6%)	
Bed size			
<350	910 (12.1%)	435 (11.6%)	<0.001
350-499	1,016 (13.5%)	593 (15.8%)	
500-750	2,904 (38.7%)	1,509 (40.3%)	
>750	2,674 (35.6%)	1,211 (32.3%)	
Location			
Rural	188 (2.5%)	17 (0.5%)	<0.001
Urban	7,316 (97.5%)	3,731 (99.5%)	
Admission timing			
Weekday	5,467 (72.9%)	2,700 (72.0%)	0.36
Weekend	2,037 (27.1%)	1,048 (28.0%)	
AMI type			
STEMI	4,085 (54.4%)	2,012 (53.7%)	0.45
Non-STEMI	3,419 (45.6%)	1,736 (46.3%)	0.45
Cardiac arrest	1,314 (17.5%)	788 (21.0%)	<0.001
Cardiogenic shock	3,497 (46.6%)	1,681 (44.9%)	0.08
Comorbidities			
Coronary artery disease	6,524 (86.9%)	3,135 (83.6%)	<0.001
Prior AMI	1,282 (17.1%)	603 (16.1%)	0.18
Prior PCI	1,704 (22.7%)	808 (21.6%)	0.17
Prior CABG	854 (11.4%)	358 (9.6%)	0.003
Dyslipidemia	4,139 (55.2%)	2,017 (53.8%)	0.18
Smoking	3,905 (52.0%)	1,928 (51.4%)	0.55
ESRD	693 (9.2%)	267 (7.1%)	<0.001
Chronic pulmonary disease	1,901 (25.3%)	908 (24.2%)	0.20
Cancer	209 (2.8%)	93 (2.5%)	0.35
Obesity	1,749 (23.3%)	824 (22.0%)	0.12
Depression	782 (10.4%)	353 (9.4%)	0.10
Dementia	289 (3.9%)	188 (5.0%)	0.004
Diabetes	2,608 (34.8%)	1,278 (34.1%)	0.49
Peripheral vascular disease	859 (11.4%)	397 (10.6%)	0.17
Chronic liver disease	415 (5.5%)	187 (5.0%)	0.23
Heart failure	3,729 (49.7%)	1,884 (50.3%)	0.57
Hypertension	3,480 (46.4%)	1,850 (49.4%)	0.003
Valvular disease	944 (12.6%)	489 (13.0%)	0.48
Prior stroke	375 (5.0%)	248 (6.6%)	<0.001

Values are mean ± SD or n (%).

AAMC = Association of American Medical Colleges; AMI = acute myocardial infarction; CABG = coronary artery bypass grafting; ESRD = end-stage renal disease; NSTEMI = non-ST-segment elevation myocardial infarction; PCI = percutaneous coronary intervention; PPI = proton pump inhibitors; STEMI = ST-segment elevation myocardial infarction.

### Medications and interventions

Famotidine and pantoprazole were the most commonly prescribed stress ulcer prophylaxis agents, and included 95.6% and 91.6% of H2B and PPIs, respectively. A similar proportion of patients were prescribed aspirin, clopidogrel, and prasugrel before intubation (all, *P* > 0.05), but those that received H2Bs received ticagrelor and cangrelor less compared to those receiving PPIs (*P* < 0.001) (Table 2). A lower proportion of patients receiving H2Bs were given vasoactive medications, any mechanical circulatory support, PCI, or renal replacement therapy before intubation (all, *P* < 0.001). The use of thrombolysis and noninvasive ventilation were similar between groups (both, *P* > 0.05).

**Table 2:** Medications and Interventions Stratified by Stress Ulcer Prophylaxis

	Stress Ulcer Prophylaxis		
	PPI (n = 7,504)	H2B (n = 3,748)	*P* Value
Medications			
IV formulation^a^	4,654 (62.0%)	2,215 (59.1%)	0.003
Median days	6 (3-12)	5 (2-9)	<0.001
Antiplatelet/anticoagulants^b^			
Aspirin	4,196 (55.9%)	2,042 (54.5%)	0.15
Plavix	2,034 (27.1%)	978 (26.1%)	0.25
Ticagrelor	2,347 (31.3%)	1,020 (27.2%)	<0.001
Prasugrel	126 (1.7%)	45 (1.2%)	0.051
Cangrelor	470 (6.3%)	186 (5.0%)	0.006
Any DAPT	3,127 (41.7%)	1,411 (37.6%)	<0.001
Heparin	6,110 (81.4%)	3,021 (80.6%)	0.29
Bivalirudin	636 (8.5%)	356 (9.5%)	0.07
Enoxaparin	347 (4.6%)	196 (5.2%)	0.16
Fondaparinux	6 (0.1%)	1 (0.0%)	0.29
Apixaban	54 (0.7%)	23 (0.6%)	0.52
Warfarin	111 (1.5%)	30 (0.8%)	0.002
Rivaroxaban	17 (0.2%)	8 (0.2%)	0.89
Dabigatran	<5 (0.0%)	<5 (0.1%)	0.75
Any vasoactive^c^ medication^b^	5,357 (71.4%)	2,506 (66.9%)	<0.001
Interventions^b^			
NIV	357 (4.8%)	170 (4.5%)	0.60
PCI	3,487 (46.5%)	1,616 (43.1%)	<0.001
Right heart catheterization	1,671 (22.3%)	684 (18.2%)	<0.001
ECMO	531 (7.1%)	126 (3.4%)	<0.001
Left heart catheterization	4,322 (57.6%)	2,017 (53.8%)	<0.001
Any MCS	2,608 (34.8%)	1,049 (28.0%)	<0.001
Intra-aortic balloon pump	1,556 (20.7%)	694 (18.5%)	0.006
Impella	937 (12.5%)	332 (8.9%)	<0.001
Renal replacement therapy	327 (4.4%)	130 (3.5%)	0.02
Thrombolysis	82 (1.1%)	42 (1.1%)	0.89

Values are median (IQR) or n (%).

DAPT = dual antiplatelet therapy; ECMO = extracorporeal membrane oxygenation; IV = intravenous; NIV = noninvasive ventilation; MCS = mechanical circulatory support; PCI = percutaneous coronary intervention; PPI = proton pump inhibitors.

a First use.

b Present before or same day as invasive mechanical ventilation.

c Norepinephrine, vasopressin, epinephrine, phenylephrine, milrinone, dobutamine, dopamine.

### Mortality

The in-hospital mortality was 41.5% (n = 1,556) for those receiving H2Bs and 43.5% (n = 3,263) for patients receiving PPIs (*P* = 0.047, Table 3). After multivariable adjustment, utilization of H2Bs compared PPIs was not associated with a difference in mortality (OR: 0.97; 95% CI: 0.89-1.06, *P* = 0.49). Adjusted results were similar when limited to patients receiving famotidine compared to pantoprazole (OR 0.97; 95% CI: 0.89-1.05, *P* = 0.46).

**Table 3:** Unadjusted Outcomes

	Stress Ulcer Prophylaxis		
	PPI (n = 7,504)	H2B (n = 3,748)	*P* Value
In-hospital mortality	3,263 (43.5%)	1,556 (41.5%)	0.047
Length of stay, days	8 (4-15)	7 (3-12)	<0.001
ICU length of stay, days	5 (2-9)	4 (2-7)	<0.001
Total cost ($)	41,718 (23,246-74,806)	33,972 (19,692-56,124)	<0.001
Discharge disposition			<0.001
Home	1,504 (20.1%)	878 (23.4%)	
Skilled nursing/rehab	1,702 (22.7%)	746 (19.9%)	
Home with services	645 (8.6%)	352 (9.4%)	
Against medical advice	42 (0.6%)	34 (0.9%)	
Expired	3,263 (43.5%)	1,556 (41.5%)	
Hospice	251 (3.3%)	129 (3.4%)	
Other	93 (1.2%)	51 (1.4%)	
Total ventilator days	4 (2-8)	4 (2-7)	<0.001
Tracheostomy	316 (4.2%)	98 (2.6%)	<0.001
Ventilator-associated pneumonia	298 (4.0%)	117 (3.1%)	0.024
*Clostridium difficile* infection	101 (1.3%)	43 (1.1%)	0.38
Blood transfusion^a^	663 (8.8%)	220 (5.9%)	<0.001
Platelet transfusion^a^	168 (2.2%)	47 (1.3%)	<0.001
Fresh frozen plasma transfusion^a^	126 (1.7%)	45 (1.2%)	0.05
Any blood product transfusion^a^	747 (10.0%)	244 (6.5%)	<0.001
EGD^a^	227 (3.0%)	8 (0.2%)	<0.001
Colonoscopy^a^	64 (0.9%)	5 (0.1%)	<0.001

Values are n (%) or median (IQR).

EGD = esophagogastroduodenoscopy; ICU = intensive care unit; PPI = proton pump inhibitors.

a Occurring after invasive mechanical ventilation.

### Secondary outcomes

The median hospital and ICU length of stays were 1 day shorter for patients receiving H2Bs (both, *P* < 0.001) (Table 3). Utilization of H2Bs remained associated with fewer hospital and ICU days after multivariable adjustment (both, *P* < 0.001). Median total ventilator days were similar between groups, but statistically lower for H2B recipients. After multivariable adjustment, H2B receipt remained associated with fewer ventilator days (IRR 0.83; 95% CI: 0.81-0.84, *P* < 0.001). H2B use was associated with decreased unadjusted and adjusted postintubation transfusions and endoscopy (EGD and colonoscopy) compared to those receiving PPIs (both, *P* < 0.001). Total hospital costs were approximately $7,746 less for patients receiving H2Bs (*P* < 0.001), which persisted after multivariable adjustment (change in estimate, −$13,454, 95% CI: −$15,442 to −$11,466, *P* < 0.001). We found that the incidence of VAP was lower in patients receiving H2Bs (3.1% vs 4.0%, *P* = 0.024), which also remained significant after multivariable adjustment (OR 0.80; 95% CI: 0.64-0.997, *P* = 0.047). We did not find a statistically significant difference in CDI between groups in unadjusted (1.1% vs 1.3%, *P* = 0.38) or adjusted analyses (OR 0.89; 95% CI: 0.62-1.27, *P* = 0.52).

### Sensitivity analysis

Utilizing variables from Tables 1 and 2 and IPTW, we did not find a difference in mortality with H2B use compared to PPI (weighted mean mortality: −0.4%; 95% CI: −2.3% to 1.5%, *P* = 0.66). Model covariates and weighted standardized differences are shown in Supplemental Table 2.

## Discussion

In this multicenter study of stress ulcer prophylaxis, we report several important findings using a largely understudied population of patients presenting with AMI. First, use of H2B compared to PPIs for stress ulcer prophylaxis did not result in a statistically significant difference in mortality after adjustment for markers of acuity. Second, patients who received stress ulcer prophylaxis with H2Bs experienced fewer days of IMV. Third, the use of H2Bs was associated with less VAP compared to those who received PPIs, but CDI was not different. Finally, patients receiving H2Bs were less likely to require postintubation transfusions and undergo endoscopy. Taken together, despite potentially more complications with PPIs, mortality was similar between groups in this patient population with AMI.

To our knowledge, this is the first study to investigate the use of PPIs vs H2Bs for stress ulcer prophylaxis in critically ill, nonsurgical patients with AMI. Patients with cardiovascular disease represent a minority in most landmark trials exploring the clinical implications of stress ulcer prophylaxis. For example, in the SUP-ICU (Stress Ulcer Prophylaxis in the Intensive Care Unit) trial, only 9% and 6% of patients had a pre-existing history of previous AMI or chronic heart failure, respectively.^18^ Similarly, in the recent PEPTIC trial, <7% of patients were reported to have a history of chronic cardiovascular disease. In addition, fewer than 10 to 15% of patients were admitted to the ICU with an acute, nonoperative cardiovascular diagnosis, which is not defined further and may represent a heterogenous group with varying management considerations.^9^ The paucity of evidence guiding stress ulcer prophylaxis strategies among critically ill patients with AMI is a gap in literature that this study addresses.

Our results parallel findings of the PEPTIC trial, which did not find a statistically significant difference in mortality among 26,000 general ICU patients receiving H2Bs compared to PPIs for stress ulcer prophylaxis.^9^ A higher mortality was reported among patients receiving PPIs in a prespecified subgroup of cardiac surgery patients, though a secondary analysis found mortality was statistically similar between treatment groups after adjusting for patient- and site-specific factors.^19^ Specific to the AMI population, prior observational studies describe an increased risk of adverse cardiovascular outcomes associated with PPI use, thought to be driven by pharmacologic interactions with dual antiplatelet therapy and endothelial dysfunction.^20^ However, one of the largest prospective randomized trials, COGENT (Clopidogrel and the Optimization of Gastrointestinal Events Trial), concluded there was no increase in major adverse cardiovascular events during a 6-month period among nonsurgical acute coronary syndrome patients coprescribed omeprazole and clopidogrel.^21^ Our findings are consistent with other studies suggesting the pharmacologic interaction between antiplatelet agents and PPIs has limited clinical impact, even in critical illness.

Our study also found that patients receiving PPIs experienced more VAP and days of IMV compared to those receiving H2Bs. PPIs promote bacterial colonization by reducing gastric acidity, which predisposes patients to VAP. By inhibiting H + ATPases, PPIs also impair neutrophil oxidative burst and phagolysosome formation, thereby blunting the immune response.^22^ Similar to our findings, Bashar et al^23^ reported a 3-fold increase in VAP among mechanically ventilated patients randomized to receive stress ulcer prophylaxis with PPIs compared to H2B, though duration of IMV was similar between the groups.

### Study limitations

Our results should be interpreted with several limitations. First, this is a retrospective analysis which utilized administrative data. However, this administrative database allowed for unique analyses, including the identification of diagnoses present on admission, date-stamped procedures, and pharmacy data. This allowed us to identify antiplatelet and anticoagulation medications relative to the start of IMV as well as reduce potential confounding by excluding patients who had a GI bleed or endoscopy before intubation. Second, we lack some clinically important data, such as laboratory, hemodynamic, and vital sign data, which would be needed to calculate severity of illness scores. Similarly, our data does not include information on enteral feeding, which may influence both the incidence of stress ulcers as well as risk of nosocomial pneumonia.^24^ Third, stress ulcer prophylaxis choice was not randomized, and our results should be hypothesis generating only. Fourth, there were notable differences between groups and residual confounding is likely. However, our sensitivity analysis using IPTW had excellent covariate balance, and results were similar. Finally, CIs were not adjusted for multiple comparisons.

## Conclusions

We found that stress ulcer prophylaxis with H2Bs compared to PPIs in patients with AMI requiring IMV was associated with fewer ventilator days and VAP; however, there was no difference in mortality between the 2 groups. Our findings suggest that choice of either stress ulcer prophylaxis, despite potentially more complications with PPIs, is associated with a similar in-hospital mortality.

PERSPECTIVES **COMPETENCY IN MEDICAL KNOWLEDGE:** Stress ulcer prophylaxis with H2Bs is associated with less ventilator days and ventilator-associated pneumonia among patients with acute myocardial infarction requiring invasive mechanical ventilation. However, mortality among patients receiving H2Bs and PPIs was not statistically different.

**TRANSLATIONAL OUTLOOK:** Future randomized studies are needed to identify an optimal stress ulcer prophylaxis strategy in critically ill patients with acute myocardial infarction.

## Funding support and author disclosures

The authors have reported that they have no relationships relevant to the contents of this paper to disclose.
