# Supplementary material for: Stress Ulcer Prophylaxis in Mechanically Ventilated Patients With Acute Myocardial Infarction
Source: JACC Adv. 2023 Dec 6;3(1):100750. doi: 10.1016/j.jacadv.2023.100750 (PMC11198220; doi:10.1016/j.jacadv.2023.100750)
Supplement: Supplemental Tables 1 and 2 [file mmc1.pdf]

Supplemental Table 1. Procedural International Classification of Diseases, Tenth Revision, Clinical Modification codes

Procedures	ICD-10 codes
CABG	0210093, 0210098, 0210099, 021009, 021009F, 021009, 02100A3, 02100A8, 02100A9, 02100AC, 02100AF, 02100AW, 02100J3, 02100J8, 02100J9, 02100JC, 02100JF, 02100JW, 02100K3, 02100K8, 02100K9, 02100KC, 02100KF, 02100KW, 02100Z3, 02100Z8, 02100Z9, 02100ZC, 02100ZF, 0210493, 0210498, 0210499, 021049C, 021049F, 021049W, 02104A3, 02104A8, 02104A9, 02104AC, 02104AF, 02104AW, 02104J3, 02104J8, 02104J9, 02104JC, 02104JF, 02104JW, 02104K3, 02104K8, 02104K9, 02104KC, 02104KF, 02104KW, 02104Z3, 02104Z8, 02104Z9, 02104ZC, 02104ZF, 0211093, 0211098, 0211099, 021109C, 021109F, 021109W, 02110A3, 02110A8, 02110A9, 02110AC, 02110AF, 02110AW, 02110J3, 02110J8, 02110J9, 02110JC, 02110JF, 02110JW, 02110K3, 02110K8, 02110K9, 02110KC, 02110KF, 02110KW, 02110Z3, 02110Z8, 02110Z9, 02110ZC, 02110ZF, 0211493, 0211498, 0211499, 021149C, 021149F, 021149W, 02114A3, 02114A8, 02114A9, 02114AC, 02114AF, 02114AW, 02114J3, 02114J8, 02114J9, 02114JC, 02114JF, 02114JW, 02114K3, 02114K8, 02114K9, 02114KC, 02114KF, 02114KW, 02114Z3, 02114Z8, 02114Z9, 02114ZC, 02114ZF, 0212093, 0212098, 0212099, 021209C, 021209F, 021209W, 02120A3, 02120A8, 02120A9, 02120AC, 02120AF, 02120AW, 02120J3, 02120J8, 02120J9, 02120JC, 02120JF, 02120JW, 02120K3, 02120K8, 02120K9, 02120KC, 02120KF, 02120KW, 02120Z3, 02120Z8, 02120Z9, 02120ZC, 02120ZF, 0212493, 0212498, 0212499, 021249C, 021249F, 021249W, 02124A3, 02124A8, 02124A9, 02124AC, 02124AF, 02124AW, 02124J3, 02124J8, 02124J9, 02124JC, 02124JF, 02124JW, 02124K3, 02124K8, 02124K9, 02124KC, 02124KF, 02124KW, 02124Z3, 02124Z8, 02124Z9, 02124ZC, 02124ZF, 0213093, 0213098, 0213099, 021309C, 021309F, 021309W, 02130A3, 02130A8, 02130A9, 02130AC, 02130AF, 02130AW, 02130J3, 02130J8, 02130J9, 02130JC, 02130JF, 02130JW, 02130K3, 02130K8, 02130K9, 02130KC, 02130KF, 02130KW, 02130Z3, 02130Z8, 02130Z9, 02130ZC, 02130ZF, 0213493, 0213498, 0213499, 021349C, 021349F, 021349W, 02134A3, 02134A8, 02134A9, 02134AC, 02134AF, 02134AW, 02134J3, 02134J8, 02134J9, 02134JC, 02134JF, 02134JW, 02134K3, 02134K8, 02134K9, 02134KC, 02134KF, 02134KW, 02134Z3, 02134Z8, 02134Z9, 02134ZC, 02134ZF
LHC	4A023N8, 4A020N7, 4A020N8, 4A023N8, 4A020N8, 4A023N7, 4A023N8, 4A023N7, B2000ZZ, B2000ZZ, B2001ZZ, B2001ZZ, B200YZZ, B200YZZ, B2010ZZ, B2011ZZ, B201YZZ, B2020ZZ, B2021ZZ, B202YZZ, B2030ZZ, B2031ZZ, B203YZZ, B2050ZZ, B2051ZZ, B205YZZ, B2060ZZ, B2061ZZ, B206YZZ, B2070ZZ, B2071ZZ, B207YZZ, B2080ZZ, B2081ZZ, B208YZZ, B20F0ZZ, B20F1ZZ, B20FYZZ, B2100ZZ, B2101ZZ, B210YZZ, B2110ZZ, B2111ZZ, B211YZZ, B2120ZZ, B2121ZZ, B212YZZ, B2130ZZ, B2131ZZ, B213YZZ, B2150ZZ, B2151ZZ, B215YZZ, B2160ZZ,

	B2161ZZ, B216YZZ, B2170ZZ, B2171ZZ, B217YZZ, B2180ZZ, B2181ZZ, B218YZZ, B21F0ZZ, B21F1ZZ, B21FYZZ
PCI	027034Z, 0270346, 0270356, 027035Z, 0270366, 027036Z, 0270376, 027037Z, 02703D6, 02703DZ, 02703E6, 02703EZ, 02703F6, 02703FZ, 02703G6, 02703GZ, 02703T6, 02703TZ, 02703Z6, 02703ZZ, 0270446, 027044Z, 027045Z, 027046Z, 02704D6, 02704DZ, 02704FZ, 02704T6, 02704TZ, 02704Z6, 02704ZZ, 0271346, 027134Z, 0271356, 027135Z, 0271366, 027136Z, 0271376, 027137Z, 02713D6, 02713DZ, 02713E6, 02713EZ, 02713F6, 02713FZ, 02713G6, 02713GZ, 02713T6, 02713TZ, 02713Z6, 02713ZZ, 0271446, 027144Z, 0271456, 027145Z, 0271466, 027146Z, 0271476, 027147Z, 02714D6, 02714DZ, 02714E6, 02714EZ, 02714F6, 02714FZ, 02714G6, 02714GZ, 02714T6, 02714TZ, 02714Z6, 02714ZZ, 0272346, 027234Z, 0272356, 027235Z, 0272366, 027236Z, 0272376, 027237Z, 02723D6, 02723DZ, 02723E6, 02723EZ, 02723F6, 02723FZ, 02723G6, 02723GZ, 02723T6, 02723TZ, 02723Z6, 02723ZZ, 0272446, 027244Z, 0272456, 027245Z, 0272466, 027246Z, 0272476, 027247Z, 02724D6, 02724DZ, 02724E6, 02724EZ, 02724F6, 02724FZ, 02724G6, 02724GZ, 02724T6, 02724TZ, 02724Z6, 02724ZZ, 0273346, 027334Z, 0273356, 027335Z, 0273366, 027336Z, 0273376, 027337Z, 02733D6, 02733DZ, 02733E6, 02733EZ, 02733F6, 02733FZ, 02733G6, 02733GZ, 02733T6, 02733TZ, 02733Z6, 02733ZZ, 0273446, 027344Z, 0273456, 027345Z, 0273466, 027346Z, 0273476, 027347Z, 02734D6, 02734DZ, 02734E6, 02734EZ, 02734F6, 02734FZ, 02734G6, 02734GZ, 02734T6, 02734TZ, 02734Z6, 02734ZZ, 02C03Z6, 02C03ZZ, 02C04Z6, 02C04ZZ, 02C13Z6, 02C13ZZ, 02C14Z6, 02C14ZZ, 02C23Z6, 02C23ZZ, 02C24Z6, 02C24ZZ, 02C33Z6, 02C33ZZ, 02C34Z6, 02C34ZZ, 02H03DZ, 02H03YZ, 02H04DZ, 02H04YZ, 02H13DZ, 02H13YZ, 02H14DZ, 02H14YZ, 02H23DZ, 02H23YZ, 02H24DZ, 02H24YZ, 02H33DZ, 02H33YZ, 02H34DZ, 02H34YZ, X2C0361, X2C1361, X2C2361, X2C3361
IABP	5A02210, 5A02110
Impella	5A0221D, 5A0211D
ECMO	5A1522G, 5A1522H, 5A1522F, 5A15223
LVAD	02HA0QZ, 02WA0QZ
Heart Transplant	02YA0Z0
TPA	3E03317, 3E04317, 3E05017, 3E05317, 3E06317, 3E08317, 3E03017, 3E04017, 3E05017, 3E06017, 3E08017
IMV:	5A1955Z, 5A1935Z, 5A1945Z, 0BH18EZ, 0BH17E
Tracheostomy	0B110F4, 0B110Z4, 0B113F4, 0B113Z4, 0B114F4, 0B114Z4
RRT	5A1D70Z, 5A1D80Z, 5A1D90Z
VAP	J95851
CDI	A047, A0471, A0472

ICD-10 = International Classification of Diseases, Tenth Revision; CABG = coronary artery bypass grafting; LHC = Left heart catheterization; PCI = Percutaneous coronary intervention; RHC = Right heart catheterization; PAC = Pulmonary artery catheterization; IABP = Intra-aortic balloon pump; LVAD = Left ventricular assist device; ECMO = Extracorporeal membrane

oxygenation; tPA = Tissue plasminogen activator; IMV = Invasive mechanical ventilation; RRT = Renal replacement therapy; VAP = Ventilator associated pneumonia; CDI = Clostridium difficile infection

Supplemental Table 2. Model covariates and weighted standardized differences

	Standardized Differences	
	Raw	Weighted
**Demographics**		
Age	-0.031	0.002
Race	-0.068	0.001
Gender	0.000	0.002
**Admission Characteristics**		
STEMI	-0.015	0.006
Cardiogenic shock	-0.035	0.003
Out of hospital arrest	0.090	0.003
Weekday admission	-0.018	-0.003
Payer	-0.084	0.000
AAMC teaching status	-0.192	0.008
Bed size	-0.046	-0.000
Region	-0.093	-0.005
Location	0.171	0.013
**Co-morbidities**		
Coronary artery disease	-0.094	0.000
Prior MI	-0.027	-0.000
Prior CABG	-0.060	0.005
Prior PCI	-0.028	0.001
Heart failure	0.012	-0.004
Hypertension	0.060	0.002
Valvular disease	0.014	0.002
Prior stroke	0.069	0.003
Smoking history	-0.012	-0.001
Peripheral vascular disease	-0.027	0.004
ESRD	-0.077	-0.001
Chronic pulmonary disease	-0.026	0.003
Liver disease	-0.024	-0.005
Dementia	0.056	-0.001
Obesity	-0.031	-0.000
Cancer	-0.019	-0.003
**Medications before IMV**		
Any DAPT	-0.082	-0.006
Vasoactive medications	-0.098	0.004
**Interventions before IMV**		
NIV	-0.011	0.000
TPA	0.003	-0.002
LHC	-0.076	0.003
PCI	-0.067	-0.001
RHC	-0.100	0.004
RRT	-0.045	-0.002
